# Genetic mapping and identification of *Rht8-B1* that regulates plant height in wheat

**DOI:** 10.1186/s12870-023-04343-3

**Published:** 2023-06-22

**Authors:** Chunyun Zhou, Hongchun Xiong, Meiyu Fu, Huijun Guo, Linshu Zhao, Yongdun Xie, Jiayu Gu, Shirong Zhao, Yuping Ding, Yuting Li, Xuejun Li, Luxiang Liu

**Affiliations:** 1 State Key Laboratory of Crop Stress Biology in Arid Areas and College of Agronomy, Northwest A&F University, Yangling, Shaanxi 712100 China; 2 Institute of Crop Sciences, National Engineering Laboratory for Crop Molecular Breeding, Chinese Academy of Agricultural Sciences, National Center of Space Mutagenesis for Crop Improvement, Beijing, China

**Keywords:** Wheat, Plant height, *Rht8-B1*, QTL mapping, Spike compactness

## Abstract

**Background:**

Plant height (PH) and spike compactness (SC) are important agronomic traits that affect yield improvement in wheat crops. The identification of the loci or genes responsible for these traits is thus of great importance for marker-assisted selection in wheat breeding.

**Results:**

In this study, we used a recombinant inbred line (RIL) population with 139 lines derived from a cross between the mutant *Rht8-2* and the local wheat variety NongDa5181 (ND5181) to construct a high-density genetic linkage map by applying the Wheat 40 K Panel. We identified seven stable QTLs for PH (three) and SC (four) in two environments using the RIL population, and found that *Rht8-B1* is the causal gene of *qPH2B.1* by further genetic mapping, gene cloning and gene editing analyses. Our results also showed that two natural variants from GC to TT in the coding region of *Rht8-B1* resulted in an amino acid change from G (ND5181) to V (*Rht8-2*) at the 175^th^ position, reducing PH by 3.6%~6.2% in the RIL population. Moreover, gene editing analysis suggested that the height of T_2_ generation in *Rht8-B1* edited plants was reduced by 5.6%, and that the impact of *Rht8-B1* on PH was significantly lower than *Rht8-D1*. Additionally, analysis of the distribution of *Rht8-B1* in various wheat resources suggested that the *Rht8-B1b* allele has not been widely utilized in modern wheat breeding.

**Conclusions:**

The combination of *Rht8-B1b* with other favorable *Rht* genes might be an alternative approach for developing lodging-resistant crops. Our study provides important information for marker-assisted selection in wheat breeding.

**Supplementary Information:**

The online version contains supplementary material available at 10.1186/s12870-023-04343-3.

## Background

The global human population is predicted to continually expand and reach 10 billion by 2050, significantly increasing the need for the safe and reliable food production. The improvement of crops using advanced technologies provides an effective strategy to meet food production demands in the future [1]. Wheat represents one of the most important staple crops worldwide, and the identification of quantitative trait loci (QTLs) important for agronomical traits, such as plant height (PH) and spikelet compactness (SC), offers critical information to ensure food security [2].

PH is tightly associated with lodging resistance and thus influences grain yield. To date, a total of 25 reduced height genes (*Rht1*–*Rht25*) have been documented in wheat [3], and these dwarf genes have been classified into gibberellic acid (GA)-sensitive or -insensitive based on response to GA treatment. The GA-insensitive *Rht-B1b* (*Rht1*) and *Rht-D1b* (*Rht2*) genes on chromosomes 4B and 4D, which encode truncated DELLA proteins, significantly increase the harvest index, and their use in breeding resulted in the well-known ‘Green Revolution’ [4, 5]. their allelic variations such as *Rht-B1c* (*Rht3*) [6], *Rht-B1e* (*Rht11*) [7], *Rht-B1p* (*Rht17*) [8], and *Rht-D1c* (*Rht10*) [9] have been identified. Moreover, several GA-sensitive dwarf genes including *Rht8* [10, 11], *Rht12* [12], *Rht13* [13], *Rht18* [14], and *Rht24* [15] have been cloned or intensively studied in wheat. *Rht12* was located on chromosome 5A and mutations in *GA2oxA13* gene produced tall overgrowth phenotype in the *Rht12* background [12]. A missense mutation of *NB-LRR* gene in *Rht13* caused height reduction [13]. The dominant *Rht18* gene was identified by isolating and sequencing chromosome 6A of overgrowth mutants, and the dwarf phenotype of this mutant was found to be caused by the increased expression of *GA2oxA9* resulting in a reduction of active GA content [14]. Map-based cloning suggested that *GA2oxA9* was the causal gene of *Rht24*, which affected GA homeostasis and led to plant height reduction [15].

Since the *Rht8* gene does not influence coleoptile length, it well complements *Rht-B1b* and *Rht-D1b* weakness, and it has been widely used in wheat breeding for several decades [16, 17]. *Rht8* was mapped on the short arm of chromosome 2D, and the SSR marker *Xgwm261* was regarded as a perfect diagnostic marker for *Rht8* previously [17, 18]. Using two wheat mutants *Rht8-2* and *Rht8-3* for construction of segregation populations, the *Rht8* gene was cloned recently, and it was found to encode an RNase H-like protein that affects bioactive GA content and changes plant height [10]. Similar results were obtained simultaneously in a map-based cloning study using a wheat variety containing the *Rht8* gene [11], and both studies also showed that the dwarf allele of *Rht8* was positively selected during wheat breeding [10, 11].

Previous analyses of near isogenic lines (NILs) [19] and transgenic plants [11] have suggested that *Rht8* not only reduces PH but also significantly decreases spike length (SL) and thus increases SC. The modification of SL or SC plays an important role in the improvement of yield potential in wheat [20, 21], and identification of associated QTLs is critical for wheat improvement. In hexaploid wheat, spike morphology is regulated by three major genes, namely *Q*, *C* (*Compactum*), and *S* (*Sphaerococcum*) located on chromosomes 5A, 2D, and 3D, respectively [22–24]. These genes exert pleiotropic effects on SC and SL, PH, and grain shape. To date, a large number of QTLs associated with spike morphology have been identified on nearly all wheat chromosomes [25]. In addition, the *VRN*, *Ppd*, and *Eps* genes have also been found to be involved in SL and development and to affect SC in wheat [26–28].

In the present study, we dissected the genetic control of PH and SC by performing QTL mapping using a recombinant inbred line (RIL) population derived from a cross between the wheat variety ND5181 and the mutant line *Rht8-2* and identified natural variation in the homoeologous gene *Rht8* that contributes to PH change for *qPH2B.1*. The linkage markers and genes influencing PH and SC identified here can be applied to molecular breeding and the promotion of wheat production.

## Materials and methods

### Plant materials and phenotypic evaluation

We established an F_7_ RIL population comprising 139 lines derived from a cross between wheat cultivar NongDa5181 (ND5181) and the mutant *Rht8-2* using the single seed descent method. The RIL population and the parents were planted in the Zhongpuchang and Changping experimental fields at the Institute of Crop Sciences, Chinese Academy of Agricultural Sciences (39°97′N, 116°34′E; Beijing, China) during the 2021–2022 crop season. Each genotype was planted in one row (15 seeds per row), which was 1.5 m long with a row spacing of 0.3 m. PH and SC (calculated by dividing spikelet number per spike by the SL) were measured at maturity with eight replicates for each line [29].

### Genotyping and QTL mapping

Genomic DNA of the RIL population and parent lines was extracted and assessed as previously described [29]. The DNA samples were hybridized to the GenoBaits Wheat 40 K Panel containing 202,971 markers. Genotyping was performed at the MOLBREEDING (Shijiazhuang) Biotech Co., Ltd. (http://www.molbreeding.com). A total of 15,258 homozygous SNPs were selected from ND5181 and *Rht8-2* for follow-up analyses. The BIN and MAP functions of IciMapping 4.1 were used to remove redundant markers and construct the genetic map, respectively. The genetic linkage map contained 1847 Bin markers across 21 chromosomes. The threshold of the logarithm of odds (LOD) score was set to 2.5, and the Kosambi map function was used to calculate the map distance from recombination frequencies. Composite interval mapping (ICIM) on IciMapping 4.1 was selected to identify QTLs for PH and SC. The mean values of the phenotypic traits in each line were used for QTL analysis. QTLs detected in two environments were regarded as stable QTLs.

### KASP marker development and QTL validation

We developed the KASP assay based on the SNPs identified within or around ND5181 and *Rht8-2* from the 40 K SNP array genotyping data. The online primer design pipeline PolyMarker (http://polymarker.tgac.ac.uk/) was used to design specific primers. The KASP assays were performed as previously described [29]. End-point fluorescence data were screened using the microplate reader FLUOstar Omega SNP (BMG LABTECH, Germany) and analyzed by the Klustering Caller Software. The KASP markers were tested on the two parents, and then the developed polymorphic KASP markers were used for the identification of genotypes in the F_7_ RIL population. QTL analysis was conducted using IciMapping 4.1.

### Cloning and sequencing of ***Rht8-B1***

Gene-specific primers were designed to amplify the full-length sequence of *Rht8-B1*. The genomic DNA of ND5181 and *Rht8-2* was extracted and used as a template in PCR. The components of each 20 µL reaction were as follows: 10 µL buffer, 4 µL dNTPs, 1 µL genomic DNA, 0.8 µL forward primer, 0.8 µL reverse primer, 0.4 µL KOD FX, and 3 µL ddH_2_O. The reaction conditions were 94 ℃ for 2 min, followed by 32 cycles of 98 ℃ for 10 s, annealing at 65 ℃ for 20 s, and 68 ℃ for 2 min, with a final extension of 68 ℃ for 5 min. The PCR products were sequenced at the Shanghai Sangon Biotech Co., Ltd. (https://www.sangon.com/).

### Generating ***Rht8-B1*** and ***Rht8-D1*** mutants by gene editing

For CRISPR/Cas9-based gene editing, single guide RNA (sgRNA) target sequences were designed and plant transformations were performed as previously described [10]. The sgRNA sequence targeting *Rht8* genes was 5’-GCCGCCGGAGAGCAGCTGCC-3’. We sequenced the *Rht8* genes in T_2_ plants and validated the mutations produced by CRISPR/Cas9-based gene editing. Single mutants of *Rht8-B1* and *Rht8-D1* were successfully selected, and the heights of edited and wild-type (WT) plants recorded and compared.

### Quantitative RT-PCR

Quantitative RT-PCR was performed according to our previous report [30]. Briefly, total RNA was isolated from the first internode below the spike using TRNzol-A^+^ Reagent (Tiangen Biotech), and then purified using an RNA purification kit (Tiangen). The first-strand cDNA was synthesized using the iScript cDNA synthesis kit (Bio-Rad), and the SsoFast EvaGreen Supermix Kit (Bio-Rad) was used for quantitative RT-PCR. This experiment was conducted on a CFX 96 Real-Time System (Bio-Rad) following the manufacturer’s instructions. The actin gene was used as an internal control. The primers used for quantitative RT-PCR are listed in Supplementary Table 5.

### Distribution analysis of ***Rht8-B1b*** in wheat accessions

A total of 305 worldwide accessions with genotypic information obtained from the Wheat Union Database (http://wheat.cau.edu.cn/WheatUnion/) were used for analysis of allelic variation in *Rht8-B1* [31–34]. The frequency of *Rht8-B1b* in wheat accessions from different geographical regions was calculated according to the number of accessions carrying *Rht8-B1b* allele.

### Accession numbers

Sequence data of *Rht8-B1a* and *Rht8-B1b* have been deposited in the GenBank data library under the accession numbers OQ512875 and OQ512876, respectively.

### Statistical analysis

Statistical analyses, namely Student’s *t*-tests and correlation analyses, were performed using SPSS v21.0 software (IBM, USA).

## Results

### Phenotypic variation of two parent lines and the RIL population

**Fig. 1:**
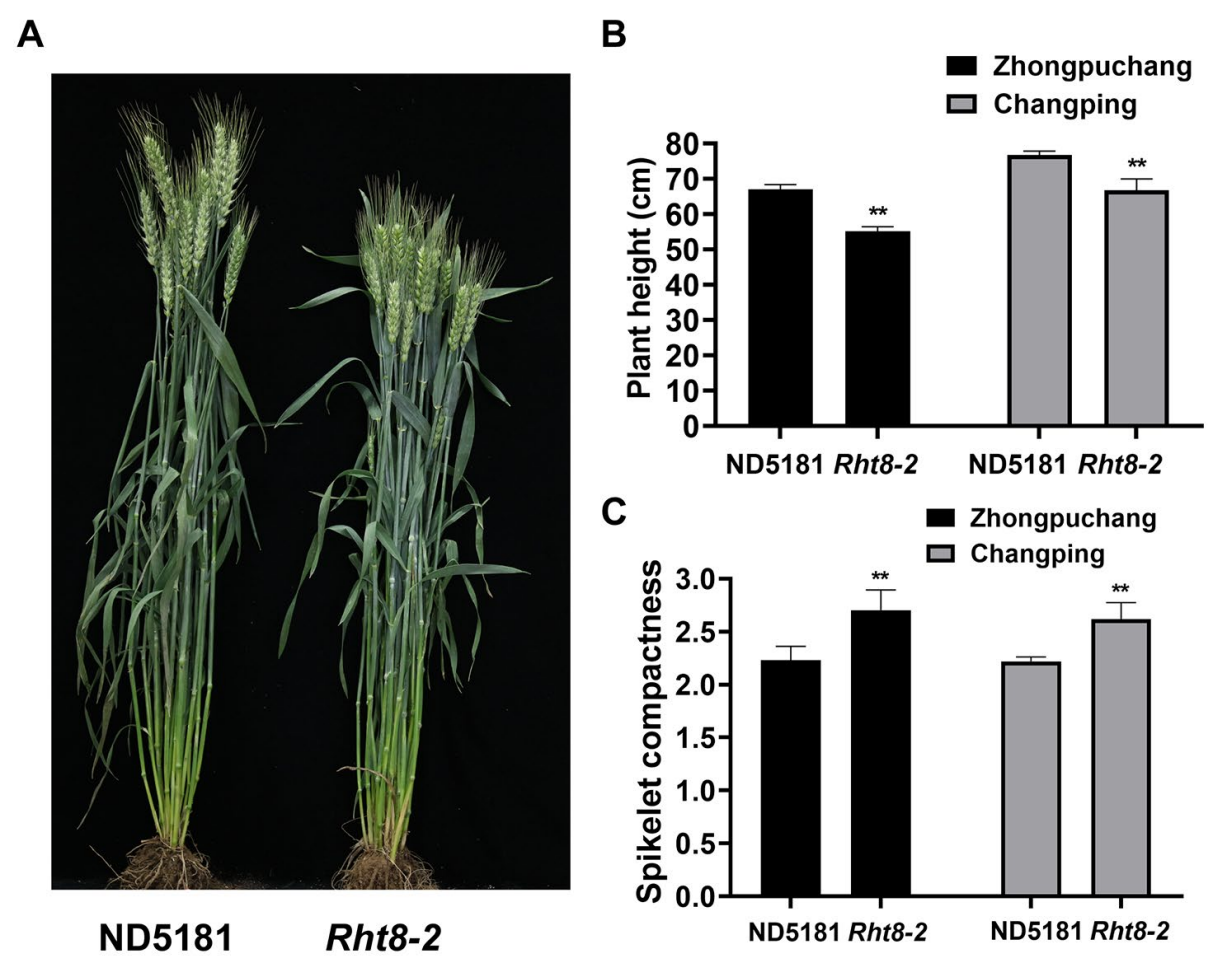
Phenotype comparisons between ND5181 and *Rht8-2*. **(A)** Phenotype of ND5181 and *Rht8-2*. **(B)** Plant height of ND5181 and *Rht8-2*. **(C)** Spikelet compactness of ND5181 and *Rht8-2*

**Fig. 2:**
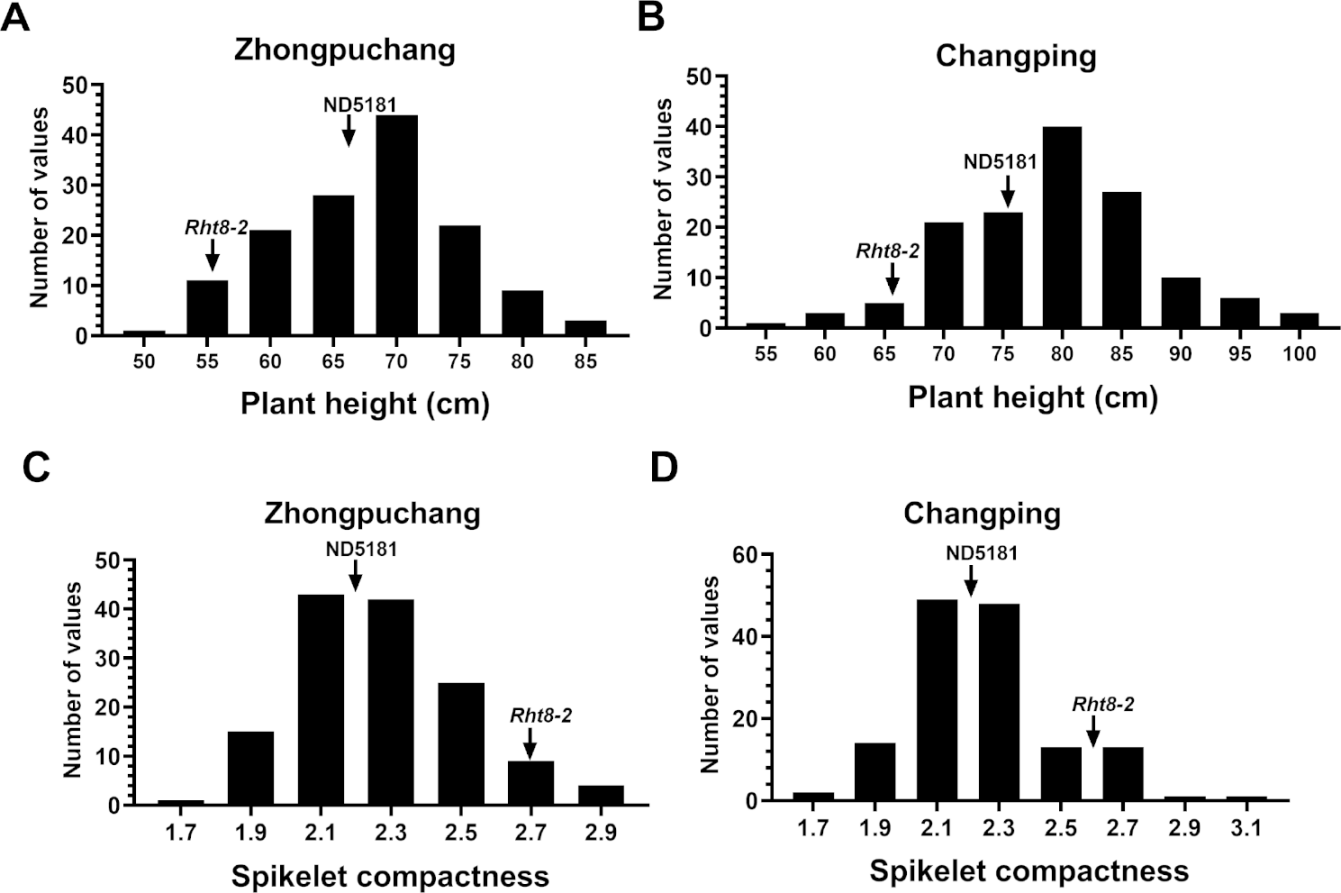
The frequency distributions of plant height, and spikelet compactness in the RIL population. **A-B**. Frequency distribution of plant height in the Zhongpuchang **(A)** and Changping **(B)** environments. **C-D**. Frequency distribution of spikelet compactness in in the Zhongpuchang **(C)** and Changping **(D)** environments. Phenotypic values of the two parental lines are marked by vertical arrows

We have previously identified a semi-dwarf wheat mutant line *Rht8-2* with high yield potential [10]. To explore QTLs associated with important agronomic traits, we constructed a RIL population including 139 lines derived from a cross between the wheat variety ND5181 and the mutant line *Rht8-2*. The two parent lines showed significant differences in PH and SC in two environments (Zhongpuchang and Changping) (Fig. 1A). Specifically, compared with ND5181, the PH of *Rht8-2* was 11 cm shorter, and SC was larger by 0.45 (Fig. 1; Table S1). In the RIL population, PH and SC displayed obvious transgressive segregation. PH and SC showed normal distributions in the two environments, suggesting they are controlled by multiple genes (Fig. 2). Pairwise correlation analysis between PH and SC showed correlation coefficients of -0.45 and -0.41 with statistical significance in the Zhongpuchang and Changping field experiments, indicating that a negative correlation between PH and SC in the RIL population.

### QTL mapping analysis

**Fig. 3:**
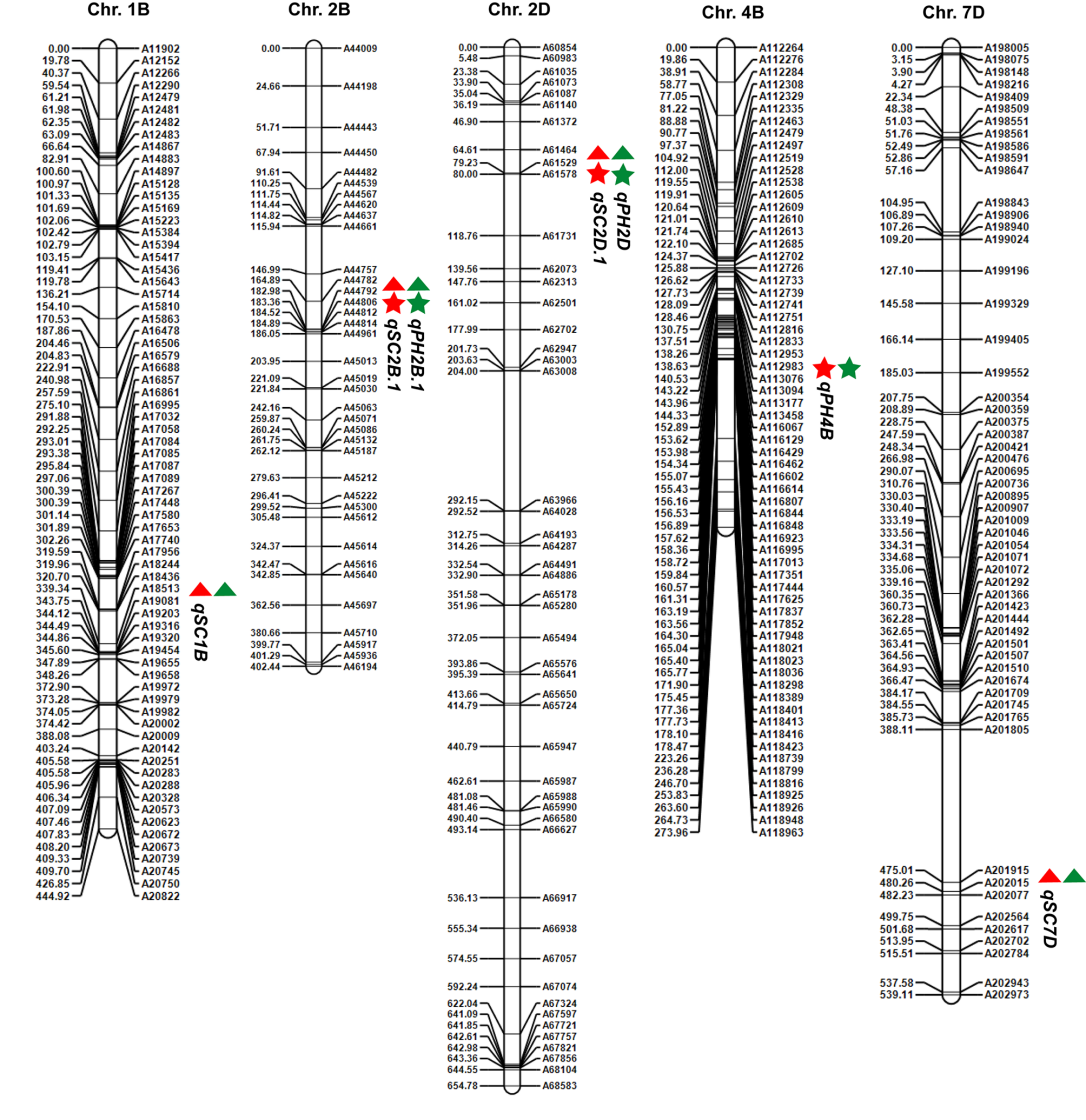
Chromosomal locations of the identified stable QTLs associated with PH and SC. The stars and triangles represent PH and SC, respectively. The red and green colors indicate data from the Zhongpuchang and Changping environments, respectively

A total of 23 QTLs associated with PH and SC were detected in the two environments on chromosomes 1A, 1B, 2B, 2D, 3A, 3B, 4B, 5A, 5B, 6B, 6D, 7A, 7B, and 7D. Among these QTLs, *qPH2B.1*, *qPH2D*, *qPH4B*, *qSC1B*, *qSC2B.1*, *qSC2D.1*, and *qSC7D*, were detected in both environments (Fig. 3; Table 1).

**Table 1:** QTLs associated with plant height (PH) and spikelet compactness (SC) in the Zhongpuchang and Changping environments identified with IciMapping 4.1

Environment	Trait Name	QTL	Chromosome	Left Marker	Right Marker	LOD	PVE (%)	Add
**Zhongpuchang**	**PH**	***qPH2B.1***	2B	A44806	A44812	11.4684	7.994	2.5902
		*qPH2B.2*	2B	A48303	A48463	8.2893	5.5607	2.1144
		***qPH2D***	2D	A61464	A61529	22.651	19.2436	4.0568
		*qPH3A*	3 A	A69302	A69519	5.8247	3.943	-1.7645
		***qPH4B***	4B	A112983	A113076	30.1221	29.7012	-4.901
		*qPH7B.1*	7B	A193948	A194156	10.2915	6.9504	2.414
		*qPH7B.2*	7B	A194789	A194814	3.9033	2.449	-1.4358
	**SC**	***qSC1B***	1B	A18513	A19081	5.0487	3.7798	0.0687
		***qSC2B.1***	2B	A44792	A44806	24.5623	24.9755	-0.1801
		*qSC2B.2*	2B	A45030	A45063	11.673	9.482	0.1119
		***qSC2D.1***	2D	A61140	A61372	13.6018	13.006	-0.1288
		*qSC2D.2*	2D	A64193	A64287	5.4426	3.9538	-0.0703
		*qSC7B*	7B	A193641	A193948	3.9214	2.7592	-0.058
		***qSC7D***	7D	A202015	A202077	2.7953	1.9425	0.0493
**Changping**	**PH**	***qPH2B.1***	2B	A44792	A44806	4.0065	2.1155	1.6056
		*qPH2B.3*	2B	A56299	A56338	5.2987	2.8879	1.9184
		***qPH2D***	2D	A61464	A61529	23.5792	17.8166	4.7225
		*qPH3B*	3B	A94610	A94625	4.2256	2.2634	-1.6462
		***qPH4B***	4B	A112816	A112833	40.1532	41.7687	-7.0218
		*qPH5A*	5 A	A133195	A133216	7.5183	4.2383	2.6582
		*qPH5B*	5B	A134432	A141070	5.1174	3.5318	-2.0132
		*qPH7A*	7 A	A179460	A179521	6.4762	3.6198	2.132
	**SC**	*qSC1A*	1 A	A7344	A7362	4.4992	4.6783	-0.0555
		***qSC1B***	1B	A18513	A19081	6.2316	6.9554	0.0686
		***qSC2B.1***	2B	A44757	A44782	12.7453	14.9828	-0.1018
		***qSC2D.1***	2D	A61578	A61731	19.362	27.6476	-0.1415
		*qSC6B*	6B	A161877	A161905	3.8673	3.9563	0.0521
		*qSC6D*	6D	A174738	A174762	3.3927	3.504	0.0514
		*qSC7B*	7B	A192944	A193088	3.8252	3.8496	-0.0502
		***qSC7D***	7D	A202015	A202077	5.3974	5.6152	0.0617

Add indicates additive effect of the ND5181 allele

Three stable QTLs associated with PH were identified on chromosomes 2B, 2D, and 4B in both environments. The major QTL *qPH4B* showed the highest LOD scores (30.1 in Zhongpuchang and 40.2 in Changping) and explained 29.7% and 41.8% of the phenotypic variation, respectively. *qPH2D* had LOD scores 22.7 (Zhongpuchang) and 23.6 (Changping) and accounted for 19.2% and 17.8% of the phenotypic variation, respectively, and *qPH2B.1* had LOD scores of 11.5 (Zhongpuchang) and 4.0 (Changping) and explained 7.9% and 2.1% of the phenotypic variation, respectively. The *qPH4B* allele from ND5181 decreased PH, while the alleles of *qPH2D* and *qPH2B.1* from *Rht8-2* reduced PH (Table 1).

We identified four stable QTLs associated with SC on chromosomes 1B, 2B, 2D, and 7D in the two environments. *qSC2B.1* had LOD scores 24.6 and 12.7 and explained 25.0% and 15.0% of the phenotypic variation in Zhongpuchang and Changping, respectively; *qSC2D.1* had LOD scores of 13.6 and 19.4 and explained 13.0% and 27.6% of the phenotypic variation, respectively; *qSC1B* had LOD scores of 5.0 and 6.2 and explained 3.8% and 7.0% of the phenotypic variation, respectively; and *qSC7D* had LOD scores of 2.8 and 5.4 and explained 1.9% and 5.6% of the phenotypic variation, respectively. Among these QTLs in ND5181, the *qSC1B* and *qSC7D* alleles increased SC and the *qSC2B.1* and *qSC2D.1* alleles decreased SC (Table 1).

### ***Rht8-B1*** is the candidate gene of ***qPH2B.1***

**Fig. 4:**
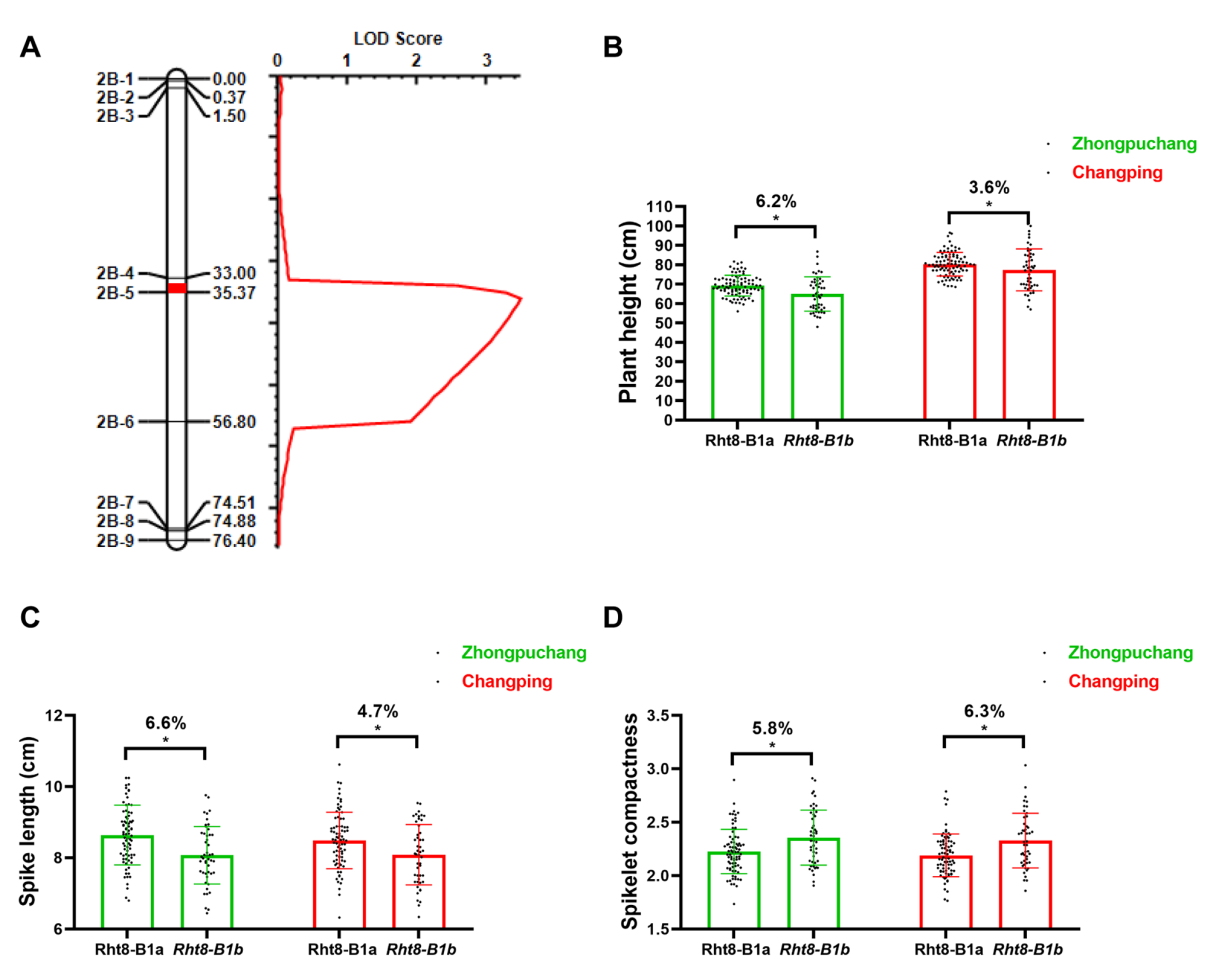
Validation of *qPH2B.1* and the predicted effects of *Rht8-B1b* in the RIL population. **(A)**. Validation of *qPH2B.1*. **B-D**. The effects of *Rht8-B1b* on plant height **(B)**, spike length **(C)**, and spikelet compactness **(D)**

To validate the mapped region of *qPH2B.1*, we successfully developed nine KASP makers around the *qPH2B.1* region based on the results of 660 K SNP array analysis between the ND5181 and *Rht8-2* varieties. These markers validated this region and delimited it to a physical interval of 3.5 Mb between markers *2B-4* and *2B-5*. This QTL showed a LOD score of 3.4 and explained 11.8% of the observed phenotypic variation (Fig. 4). Given that this region included *Rht8-B1* (TraesCS2B02G073600), a homoeologous gene of *Rht8*, we sequenced the region and uncovered two genetic variants (39,418,567–39,418,568, GC in ND5181 and TT in *Rht8-2*) in the coding region of the gene, which resulted in an amino acid change from G (ND5181) to V (*Rht8-2*) at the 175^th^ position. We then analyzed the effects of *Rht8-B1* on PH and SC in the RIL population and found that TT allele reduced PH by 6.2% and 3.6%, shortened SL by 6.6% and 4.7%, and increased SC by 5.8% and 6.3% in the Zhongpuchang and Changping field experiments, respectively (Fig. 4).

### ***Rht8-B1*** exhibited a lower impact on PH reduction than ***Rht8-D1***

**Fig. 5:**
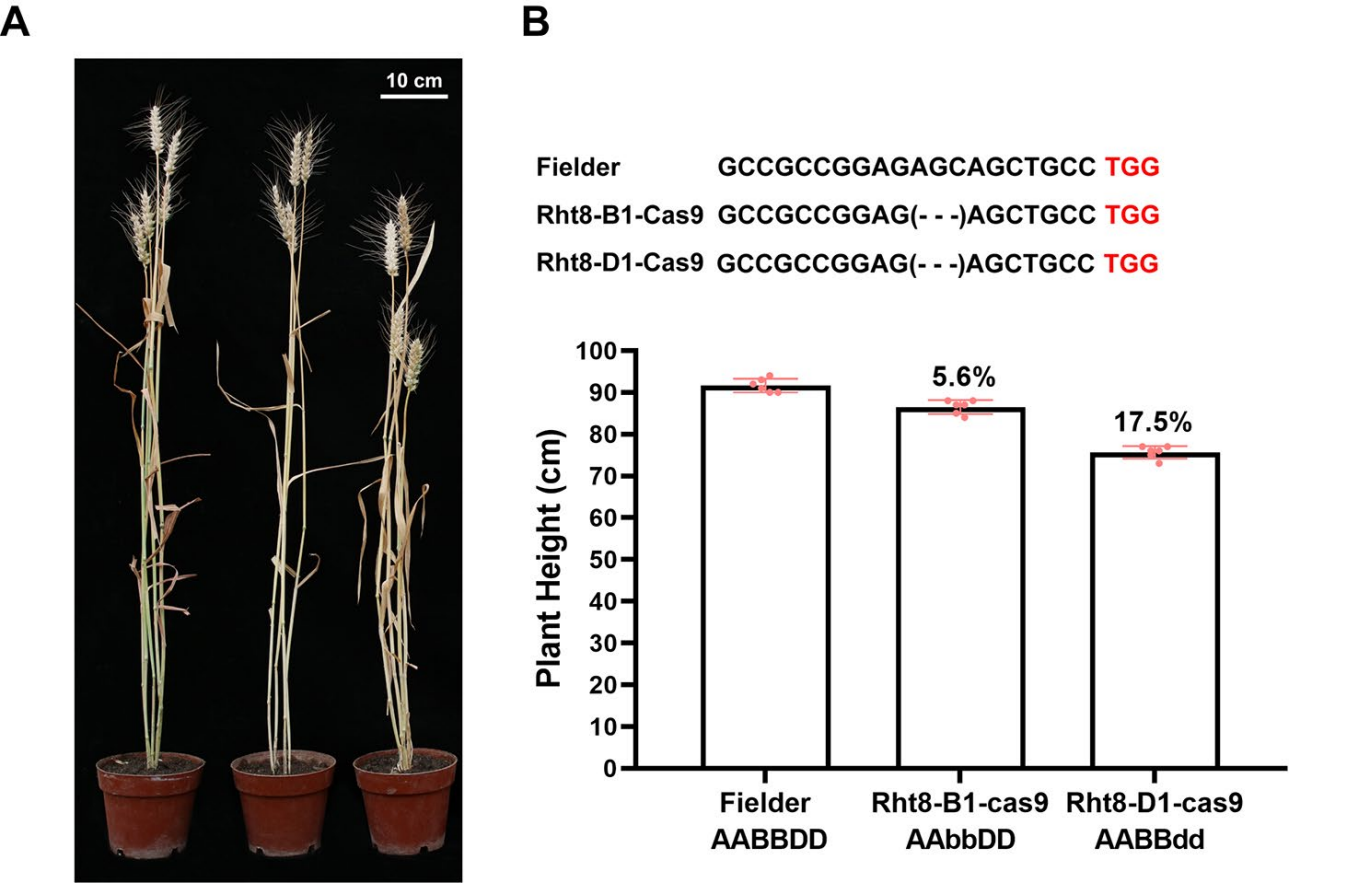
The effects of *Rht8-B1* and *Rht8-D1* on plant height of CRISPR-Cas9-edited plants. **(A)** Phenotype of Fielder and edited lines (*Rht8-B1* and *Rht8-D1*). **(B)** Plant height of Fielder and edited lines (*Rht8-B1* and *Rht8-D1*)

We compared the effects of *Rht8-B1* and the previously reported *Rht8-D1* gene on PH by knocking out both genes in a Fielder background and evaluating the PH of the T_2_ lines. The results showed that the PH of edited plants was significantly lower than that of control plants. Specifically, edited *Rht8-B1* and *Rht8-D1* plants showed a PH reduction of 5.6% and 17.5%, respectively, compared with the WT control, suggesting that *Rht8-B1* had a smaller effect on PH than *Rht8-D1* (Fig. 5; Figure S1).

### Comparison of the expression of ***Rht8-B1*** and ***Rht8-D1***

**Fig. 6:**
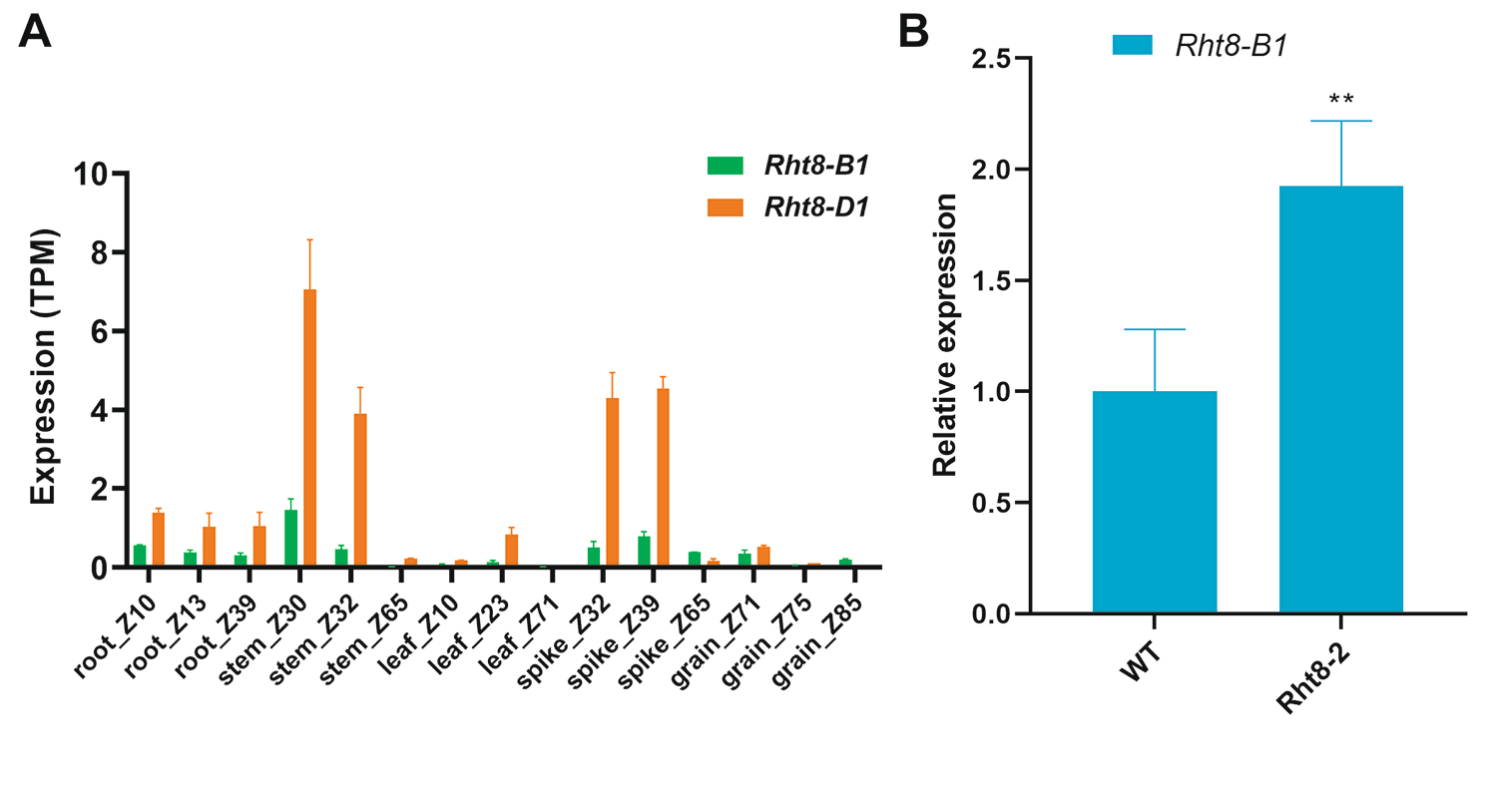
**A**. Expression data for *Rht8-B1* and *Rht8-D1* from the Hexaploid Wheat Expression Database. **B**. Analysis of *Rht8-B1* expression in the first internode below the spike in WT and *Rht8-2*

To examine the difference in expression patterns between *Rht8-B1* and *Rht8-D1*, we analyzed their transcript levels using the Hexaploid Wheat Expression Dataset [35] and found that although both genes were highly expressed in the stem at the jointing stage, the expression of *Rht8-D1* was significantly higher than that of *Rht8-B1* (Fig. 6A). Our previous study suggested that a frameshift mutation in *Rht8-D1* caused the dwarfism phenotype in *Rht8-2* and that its expression was significantly lower in the mutant compared with that in WT [10]. To investigate the effects of mutation of *Rht8-D1* on the B subgenome of *Rht8*, we analyzed the expression of *Rht8-B1* in the first internode below the spike of the mutant *Rht8-2* and WT. The results showed that the expression of *Rht8-B1* was remarkably higher in *Rht8-2* (Fig. 6B), indicating that the mutation of *Rht8-D1* affected the transcript level of *Rht8-B1*.

### Distribution of ***Rht8-B1*** in wheat varieties worldwide

**Fig. 7:**
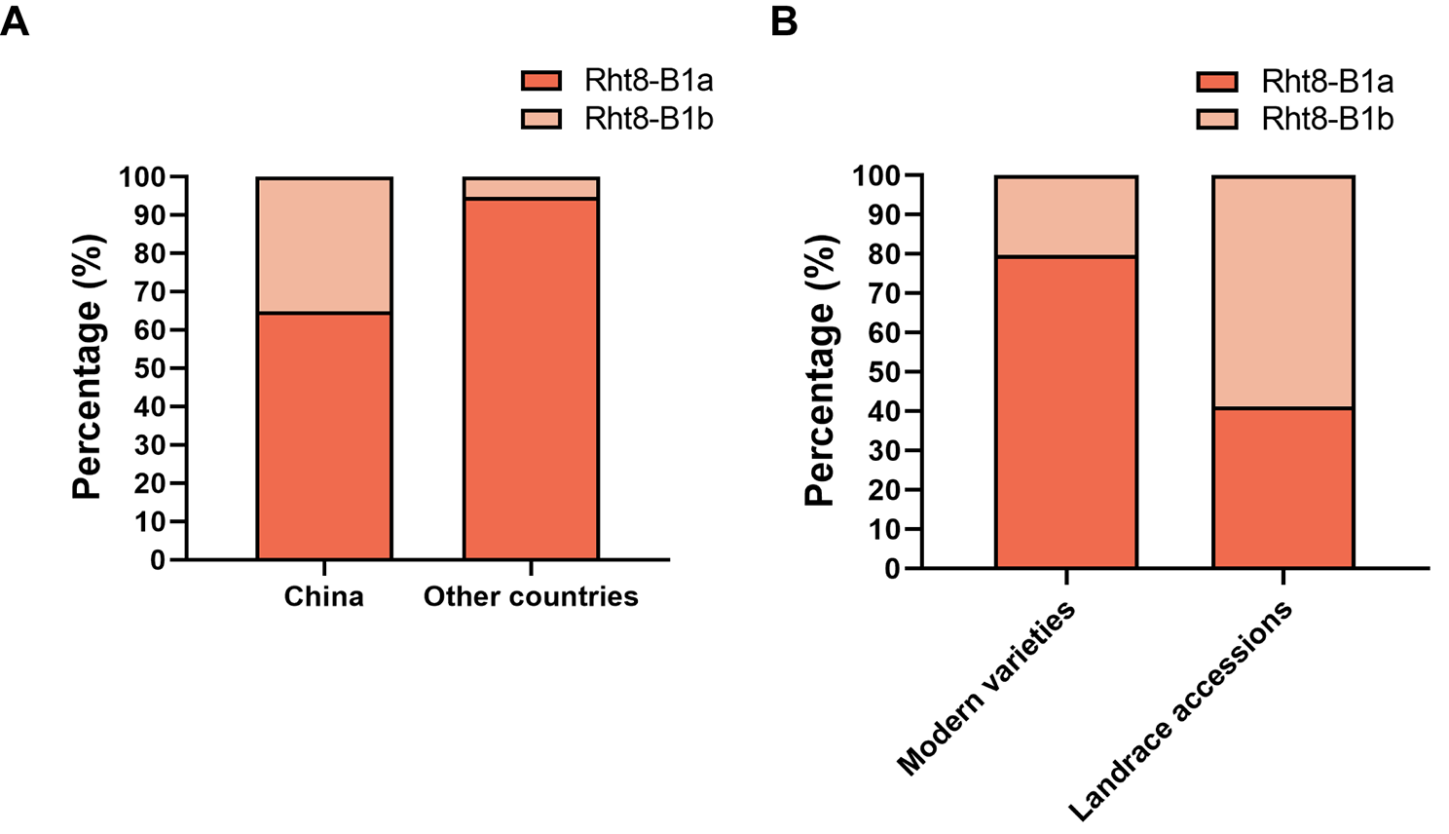
Analysis of the distribution of *Rht8-B1b* in wheat accessions worldwide. GC and TT represent *Rht8-B1a* and *Rht8-B1b*, respectively

We used a total of 305 worldwide accessions from the Wheat Union Database (http://wheat.cau.edu.cn/WheatUnion/), namely 193 accessions from China and 112 accessions from other countries, for the analysis of *Rht8-B1* allelic variation. We found that 68 Chinese accessions (35.2%) contained the *Rht8-B1b* TT allele, compared with only 6 accessions (5.4%) in other countries (Fig. 7). Of the Chinese accessions, 20.3% of the 118 modern varieties and 58.7% of the 75 landrace accessions had *Rht8-B1b* allele, (Table S2). These results suggest that *Rht8-B1b* allele was not widely used historically for wheat breeding.

## Discussion

Semi-dwarf wheat usually possesses high lodging resistance with high yield stability [4]. The identification of novel genes regulating PH is pivotal for improving lodging resistance through wheat breeding. In this study, we identified *Rht8-B1* as a novel regulator of PH by performing genetic mapping and gene editing analyses. By combining mapping using the Wheat40K array and molecular markers, *qPH2B.1* was mapped within a physical interval of 36.5–40.0 Mb between markers *2B-4* and *2B-5* on chromosome 2B of the Chinese Spring reference genome v1.0 (Fig. 4A). The *Rht8-B1* gene was located to position ~ 39.4 Mb on chromosome 2B, making it a candidate gene for *qPH2B.1*. Genotyping of *Rht8-B1* in the RIL population showed that genetic variants in this gene were associated with PH (Fig. 4B). Further gene editing of *Rht8-B1* validated its function in the regulation of PH (Fig. 5). These results suggest *Rht8-B1* is the causal gene of *qPH2B.1*.

We recently identified *Rht8* on chromosome 2D (*Rht8-D1*) by performing map-based cloning using two dwarf mutants, *Rht8-2* and *Rht8-3*; this gene encodes a Ribonuclease H-like protein that modifies PH by regulating the bioactive GA content [10]. Here, we used *Rht8-2* for the construction of a RIL population and found a major QTL – *qPH2D –* on chromosome 2D with a high LOD score, which probably represents *Rht8-D1*. In contrast to the *Rht8-D1* (*qPH2D*) locus, *qPH2B.1* had a minor effect on PH (Figs. 3 and 4 A), which is consistent with gene editing results (Fig. 5) and reports by Chai et al. [11]. The larger effect of *Rht8-D1* on PH relative to *Rht8-B1* is also in accordance with the higher expression of *Rht8-D1* in the stem (Fig. 6A). Previous studies extensively investigated the function of the *Rht8* orthologous gene *TAC4/sg2* in rice [36, 37]. A stop-gain mutation in *TAC4* was found to lead to a greater tiller angle and a dwarf phenotype that may result from changes in IAA content and distribution. In addition, an 8-bp deletion in *sg2* was found to result in a smaller grain phenotype due to repressed cell expansion in spikelet hulls and a semi-dwarf phenotype. These observations indicate that orthologous genes to *Rht8* affect multiple agronomic traits and play different roles, highlighting the need for further exploration of the effects of *Rht8-B1* and *Rht8-D1* on PH in the future.

The semidwarf *Rht8-B1b* allele has high potential for utilization in wheat breeding. By analyzing the genetic sequence of *Rht8-B1* in the mutant *Rht8-2* and WT variety Jing411, we found that the *Rht8-B1b* allele was derived from the WT, indicating that it is a natural variant. Importantly, the distribution of the *Rht8-B1b* allele in global wheat varieties suggests it has not been widely utilized historically in wheat breeding outside of China (Fig. 7). Similar to *Rht8-D1b*, the *Rht8-B1b* allele had no effects on thousand grain weight. Several studies have suggested that dwarf or semidwarf genes are associated with a decrease in thousand grain weight and ultimately affect wheat yield [38–40]. The combination of *Rht8-B1b* and other dwarf genes in wheat breeding can thus be an alternative approach for developing lodging-resistant wheat.

We found consistency between the stable QTLs associated with PH and SC detected in this study and previous reports. For example, *Rht-B1b* was located at ~ 30.8 Mb on chromosome 4B within the chromosomal region corresponding to *qPH4B* [5]; *qSC2D.1* was identified between molecular markers A61578 and A61731 in the interval 20.8–30.3 Mb, and is closely linked to *Rht8-D1*, which was reported to significantly reduce SL [11]; *qSC7D* was located between molecular markers A202015 and A202077 in the interval 584.5–588.2 Mb, and is closely linked to *WAPO1-7D*, which regulates spikelet number per spike [41]. We also identified several putatively novel QTLs for PH and SC, including *qPH7B.1* on chromosome 7BL, which had a LOD score 10.3 and explained 7.0% of the phenotypic variation in PH, and *qSC1B* located on the long arm of chromosome 1B between molecular markers A18513 and A19081, which is associated with SC.

## Conclusion

We identified seven stable QTLs for PH and SC in two environments using a RIL population. Using mapping and gene editing analyses we found that *Rht8-B1* is the causal gene of *qPH2B.1* and affects PH variation. *Rht8-B1* had less of an effect on PH than *Rht8-D1* and has not been widely utilized in wheat breeding. This implies that combining *Rht8-B1b* with other favorable *Rht* genes has great potential for breeding lodging-resistant wheat varieties. Our study revealed novel markers and QTLs, providing important information for marker-assisted selection in wheat.

## Electronic supplementary material

Below is the link to the electronic supplementary material.

Supplementary Material 1

Supplementary Material 2

Supplementary Material 3

## Data Availability

All data generated or analyzed during this study are included in the main text article and its supplementary files. The variant data for this study have been deposited in the European Variation Archive (EVA) at EMBL-EBI under accession number PRJEB60409 (http://www.ebi.ac.uk/eva/?eva-study=PRJEB60409).
