# Supplementary figures and images for: Genetic mapping and identification of Rht8-B1 that regulates plant height in wheat
Source: BMC Plant Biol. 2023 Jun 22;23:333. doi: 10.1186/s12870-023-04343-3 (PMC10286341; doi:10.1186/s12870-023-04343-3)

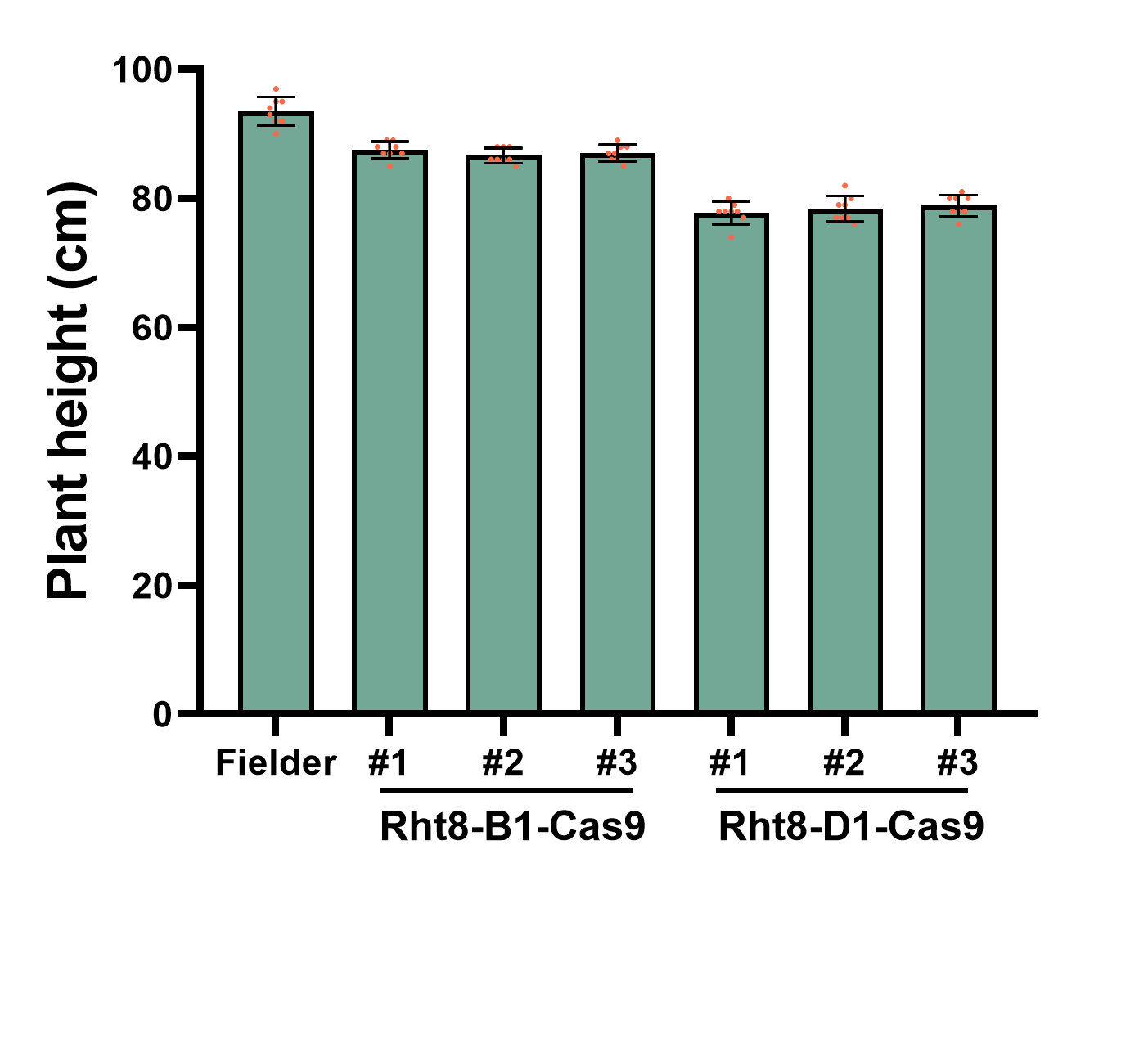

Supplement: Supplementary file 1 — Supplementary Material 1 [file 12870_2023_4343_MOESM1_ESM.tif]
