# Supplementary material for: Genetic mapping and identification of Rht8-B1 that regulates plant height in wheat
Source: BMC Plant Biol. 2023 Jun 22;23:333. doi: 10.1186/s12870-023-04343-3 (PMC10286341; doi:10.1186/s12870-023-04343-3)
Supplement: Supplementary file 3 — Supplementary Material 3 [file 12870_2023_4343_MOESM3_ESM.docx]

**Supplementary material**

**Figure S1. Plant height of *Rht8-B1* and *Rht8-D1* gene editing lines.**

**Table S1. Summary statistics for plant height and spikelet compactness for the two parental lines and the RIL population.**

**Table S2. Materials used for distribution analysis of *Rht8-B1b*.**

**Table S3. Primers used for genetic mapping of *Rht8-B1* gene.**

**Table S4. Primers used for quantitative RT-PCR.**
